# The Pregravid Vascular Risk Factor Profile of Low-Risk Women Who Develop Pregnancy Outcomes That Predict Future Cardiovascular Disease

**DOI:** 10.1089/whr.2021.0006

**Published:** 2021-03-23

**Authors:** Roslyn Mainland, Shi Wu Wen, Hongzhuan Tan, Shujin Zhou, Chang Ye, Minxue Shen, Graeme N. Smith, Mark C. Walker, Ravi Retnakaran

**Affiliations:** ^1^ Leadership Sinai Centre for Diabetes, Mount Sinai Hospital, Toronto, Ontario, Canada.; ^2^ OMNI Research Group, Department of Obstetrics and Gynecology, University of Ottawa, Ottawa, Ontario, Canada.; ^3^ Clinical Epidemiology Program, Ottawa Hospital Research Institute, Ottawa, Ontario, Canada.; ^4^ Department of Epidemiology and Community Medicine, University of Ottawa, Ottawa, Ontario, Canada.; ^5^ School of Public Health, Central South University, Changsha, China.; ^6^ Liuyang Municipal Hospital of Maternal and Child Health, Liuyang, China.; ^7^ Queen's Perinatal Research Unit, Department of Obstetrics and Gynecology, Queen's University, Kingston, Ontario, Canada.; ^8^ Division of Endocrinology, University of Toronto, Toronto, Ontario, Canada.; ^9^ Lunenfeld-Tanenbaum Research Institute, Mount Sinai Hospital, Toronto, Ontario, Canada.

**Keywords:** cardiovascular risk factors, metabolic syndrome, preconception, prepregnancy

## Abstract

***Background:*** Women with a history of certain adverse outcomes in pregnancy (preterm birth, delivery of a small-for-gestational age [SGA] infant, preeclampsia, and gestational diabetes mellitus [GDM]) have an elevated lifetime prevalence of metabolic syndrome (MetS) and cardiovascular disease, compared with their peers. However, it is not known if MetS precedes the index pregnancy in young, nulliparous women who experience these antepartum outcomes. Thus, we sought to evaluate the relationship between pregravid cardiovascular risk factor profile and these pregnancy outcomes in low-risk women.

***Methods:*** In this prospective preconception cohort study, 1183 newly married women underwent systematic assessment of cardiovascular risk factors (anthropometry, blood pressure, lipids, glucose) at median 24.7 weeks before pregnancy, whereupon they were followed for the outcomes of preterm birth, SGA delivery, preeclampsia, and GDM.

***Results:*** Women who had pregravid MetS (harmonized definition) (*n* = 49) were more likely to have a Caesarean delivery than their peers (61.4% vs. 38.6%, *p* = 0.003). However, they did not have a higher incidence of preterm delivery, SGA, preeclampsia, or GDM. Similarly, women who had at least one of these adverse pregnancy outcomes (*n* = 141) did not have a higher prevalence of MetS or any of its component disorders before pregnancy. Indeed, before pregnancy, there were no significant differences between these women and their peers in waist circumference, body mass index, blood pressure, fasting glucose, triglycerides, low-density-lipoprotein, or high-density-lipoprotein cholesterol.

***Conclusions:*** The adverse cardiovascular risk factor profile that is seen in women with a history of preterm birth, SGA, preeclampsia, or GDM does not necessarily manifest before their pregnancy.

## Introduction

Cardiovascular disease (CVD) is the leading cause of death among females in the developed world and is increasing in prevalence among women aged 35–54 years.^1,2^ Pregnancy serves as a valuable screening opportunity for young women, as certain adverse pregnancy outcomes are predictive of future cardiometabolic disease. Most notably, it is now recognized that gestational diabetes mellitus (GDM),^3–7^ preeclampsia,^8–12^ delivery of a small-for-gestational age (SGA) infant,^8,13–15^ and preterm delivery^8,9,13,16^ are each associated with maternal CVD and cardiovascular mortality later in life. The mechanism linking these pregnancy outcomes to future CVD is not certain but is believed to reflect an enhanced propensity for accruing cardiovascular risk factors. Compared to their peers, women with a history of these four adverse pregnancy outcomes have a higher prevalence of cardiovascular risk factors in the years following their pregnancies. Indeed, women who have a history of GDM, preeclampsia, preterm delivery, or delivery of an SGA infant have a higher prevalence of metabolic syndrome (MetS),^17–21^ a clinical construct that is defined by the coexistence of multiple cardiovascular risk factors within an individual. However, it is not known if this adverse cardiometabolic risk factor profile necessarily precedes the index pregnancy or develops in the years following the adverse pregnancy outcome. Thus, in this study, we sought to examine the relationship between the maternal pregravid cardiometabolic risk factor profile and these four adverse pregnancy outcomes that predict future risk of CVD (GDM, preeclampsia, preterm delivery, and delivery of an SGA infant) in a low-risk population of young, nulliparous women.

## Methods

### Study population

This was a prospective preconception cohort study conducted in the Liuyang region of Hunan, China. At the time of marriage, women in this region typically attend a premarriage health clinic at the Liuyang Maternal and Infant Hospital. Among the women attending this clinic, those who were planning to conceive in the next 6 months were asked to participate in the study. The study protocol has been previously described in detail.^22,23^ In brief, participating women underwent pregravid cardiometabolic characterization at the time of recruitment and, when they later became pregnant, were followed across the pregnancy to delivery. The current analysis was limited to those who had complete evaluation of MetS component disorders at baseline (*n* = 1183). The study has been approved by the Institutional Research Ethics Boards of Central South University (Changsha, Hunan, China), Ottawa Hospital Research Institute (Ottawa, Canada), and Mount Sinai Hospital (Toronto, Canada). All participants have provided written informed consent.

There were 3375 women recruited into the cohort, of whom 2382 completed a singleton pregnancy. Among these women, 1564 had complete delivery data. After the exclusion of those with incomplete metabolic or covariate data and women who were >5 weeks pregnant at their baseline assessment based on back-dating of gestational age at delivery, the study population for the current analysis consisted of 1183 women. Compared to the 1183 women in this analysis, the other 2192 women who were recruited were slightly younger (mean 24.6 years; *p* = 0.005), with slightly higher body mass index (BMI) (mean 20.4 kg/m^2^; *p* = 0.02) and no difference in smoking status (*p* = 0.40) (data not shown).

### Pregravid assessment

At the time of recruitment, women were asked to undergo a pregravid health assessment. Each woman completed interviewer-administered questionnaires that addressed demographics, lifestyle, and medical history. Furthermore, women underwent a cardiometabolic assessment that consisted of the following components:

(i) Anthropometrics—Height and weight were measured with a wall-mounted stadiometer and scale, with both measurements performed twice and the averages recorded. Waist circumference was measured at the midpoint between the lower edge of the ribs and the iliac crest, with two measurements performed and the average recorded.
(ii) Blood pressure—Automated noninvasive blood pressure monitors were used to measure blood pressure. Measurements were taken in the seated position after 10 minutes of rest. The average of two measurements performed 10 minutes apart was recorded.
(iii) Blood glucose and lipid profile—Women were asked to undergo an overnight fast before serum samples were drawn the next morning. Samples were put on ice immediately and transported to the Central South University central laboratory within 30 minutes. Samples were centrifuged at 4°C and 3000 rpm for 10 minutes, and then total cholesterol, high-density-lipoprotein (HDL) cholesterol, triglycerides, and glucose were measured by standard clinical biochemistry. The Friedewald equation was used to calculate low-density-lipoprotein (LDL) cholesterol.

This cardiometabolic assessment was used to identify women with pregravid MetS, as defined by the harmonized criteria of the National Heart Lung and Blood Institute, American Heart Association, International Atherosclerosis Society, International Diabetes Federation, World Heart Federation, and International Association for the Study of Obesity.^24^ By these criteria, study participants were identified with MetS if three or more of the following findings were present: (i) waist circumference ≥80 cm; (ii) systolic blood pressure ≥130 mmHg and/or diastolic blood pressure ≥85 mmHg, or use of antihypertensive drug treatment in a patient with a history of hypertension; (iii) triglycerides ≥1.7 mmol/L or drug treatment for hypertriglyceridemia; (iv) HDL cholesterol <1.3 mmol/L; or (v) fasting glucose ≥5.6 mmol/L or drug treatment for hyperglycemia.^24^

### Assessment of pregnancy outcomes

Participants were followed from baseline assessment until subsequent pregnancy, and then across pregnancy to delivery. At delivery, infant characteristics were collected, including sex, birthweight, and gestational age (based on last menstrual period). Large-for-gestational age (LGA) and SGA infants were defined as birthweight for gestational age >90th percentile and <10th percentile, respectively, based on established birthweight centiles for the Chinese population.^25^ Maternal pregnancy data was also collected, including total weight gain in pregnancy, whether or not a Caesarian section was performed, and any diagnosed maternal medical conditions, including GDM and preeclampsia. Gestational diabetes was diagnosed on 2-hour 75 g oral glucose tolerance test if one of the following thresholds were met: fasting glucose ≥5.1 mmol/L; 1-hour glucose ≥10.0 mmol/L; or 2-hour glucose ≥8.5 mmol/L. Preeclampsia was diagnosed based on blood pressure ≥140/90 mmHg and 24-hour urine protein >0.3 g or positive random urine protein, at ≥20 weeks gestation.

### Statistical analyses

**FIG. 1.**
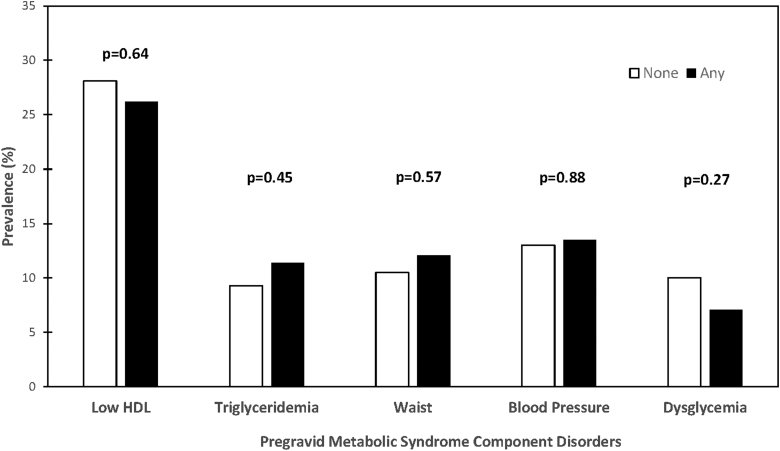
Prevalence of pregravid metabolic syndrome component disorders in women who subsequently had any of the pregnancy outcomes associated with future CV risk (“any”) and those who had none of these pregnancy outcomes (“none”). CV, cardiovascular.

**FIG. 2.**
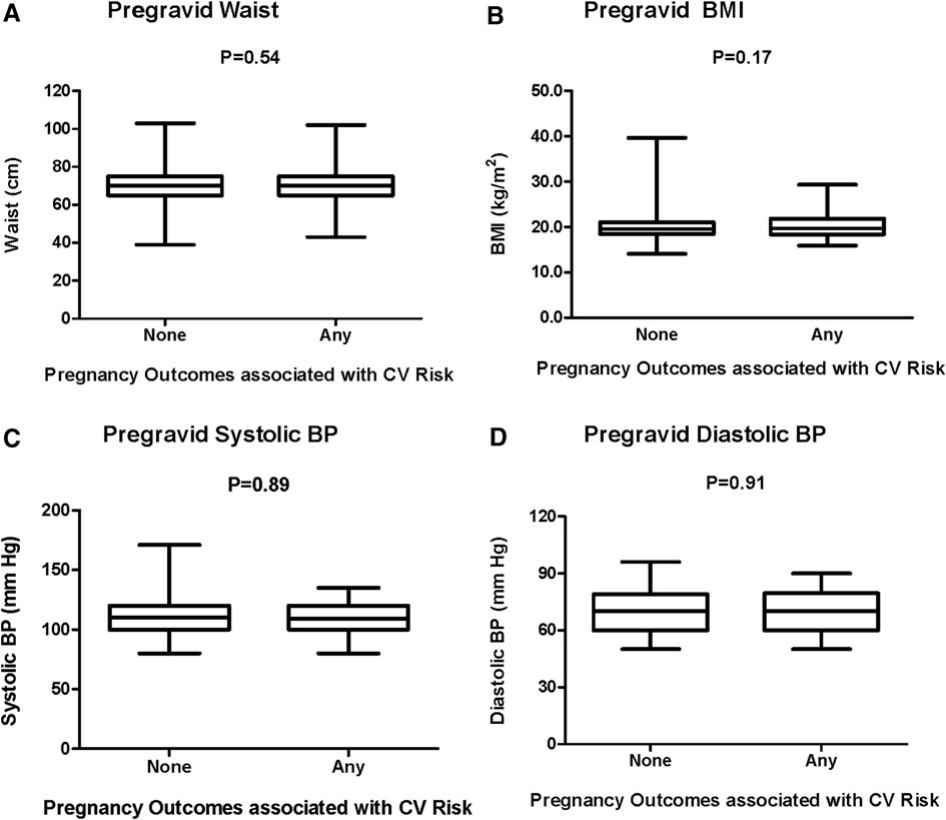

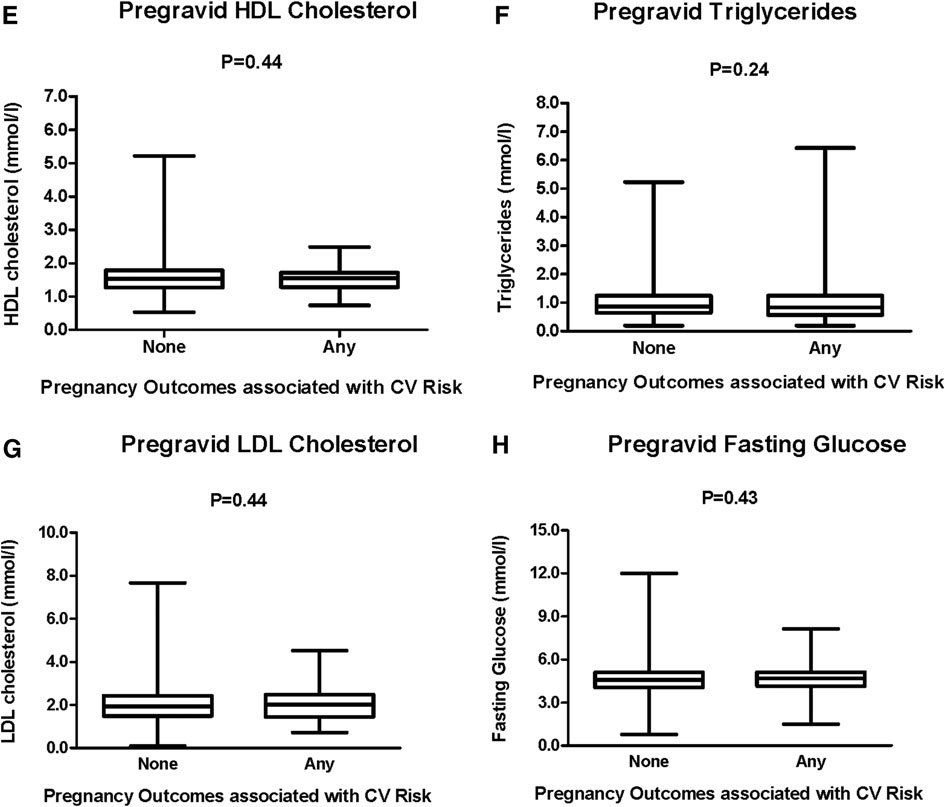
Comparison of the following pregravid CV risk factors between women who subsequently had any of the pregnancy outcomes associated with future CV risk (“any”) and those who had none of these pregnancy outcomes (“none”): **(A)** waist; **(B)** BMI; **(C)** systolic blood pressure; **(D)** diastolic blood pressure; **(E)** HDL cholesterol; **(F)** triglycerides; **(G)** LDL cholesterol; and **(H)** fasting glucose. BMI, body mass index; HDL, high-density lipoprotein; LDL, low-density lipoprotein.

All analyses were performed using SAS 9.4 (SAS Institute, Cary, NC). The study population was first stratified into two groups: women who had pregravid MetS and those who did not (No MetS). Prepregnancy demographic and clinical characteristics were compared between the two groups (Table 1), and then pregnancy outcomes were compared between the groups (Table 2). To consider unequal variances, we applied Satterthwaite method to compare those continuous variables that were normally distributed and Wilcoxon Rank-Sum nonparametric test to compare those that were skewed. Categorical variables were compared using chi-square or Fisher's exact test. To investigate whether the women who developed *any* of four pregnancy outcomes (GDM, preeclampsia, preterm delivery, or delivery of an SGA infant) associated with future CVD showed evidence of an adverse cardiovascular risk factor profile before pregnancy, the study population was then divided into two groups: women who subsequently had any of the pregnancy outcomes associated with future cardiovascular risk (“any”) and those who had none of these pregnancy outcomes (“none”). We evaluated the prevalence of each of the pregravid MetS component disorders in the two groups and compared the differences in prevalence between the groups by chi-square test (Fig. 1). Furthermore, two-sample *t*-test was performed to determine whether there were differences in pregravid cardiovascular risk factors between the groups (Fig. 2).

**Table 1.** Prepregnancy Demographic and Clinical Characteristics of Women Who Had Pregravid Metabolic Syndrome and Those Who Did Not Have

Pregravid assessment	No MetS (*n* = 1134)	MetS (*n* = 49)	*p*
Weeks before pregnancy, weeks	24.7 (7.1–67.9)	26.9 (4.3–60.7)	0.84
Age, years	24.8 ± 3.1	24.3 ± 3.5	0.34
Years of education, years	9 (9–12)	9 (9–12)	0.07
Smoking, %	0.5	0	0.99
Passive smoking exposure, %	6.7	6.1	0.99
Preexisting hypertension, %	0.3	2.0	0.16
Preexisting diabetes, %	0.2	2.0	0.12
BMI, kg/m^2^	20.2 ± 2.3	23.1 ± 3.2	<0.0001
Waist circumference, cm	70.0 ± 7.0	79.8 ± 8.5	<0.0001
Systolic blood pressure, mmHg	109.6 ± 12.8	118.6 ± 12.1	<0.0001
Diastolic blood pressure, mmHg	70.0 ± 9.1	76.4 ± 9.7	<0.0001
Total cholesterol, mmol/L	3.9 ± 1.1	4.3 ± 2.2	0.14
LDL cholesterol, mmol/L	2.0 ± 0.8	2.4 ± 1.0	0.02
HDL cholesterol, mmol/L	1.6 ± 0.4	1.1 ± 0.2	<0.0001
Triglycerides, mmol/L	0.8 (0.6–1.2)	1.9 (1.4–2.9)	<0.0001
Glucose, mmol/L	4.5 ± 1.1	5.5 ± 1.0	<0.0001

Continuous data are presented as mean ± standard deviation (if normally distributed) or median followed by interquartile range in parentheses (if skewed). Categorical data are presented as percentages.

BMI, body mass index; HDL, high-density lipoprotein; LDL, low-density lipoprotein; MetS, metabolic syndrome.

**Table 2.** Pregnancy Outcomes in Women Who Had Pregravid Metabolic Syndrome and in Those Who Did Not Have

At delivery	No MetS (*n* = 1134)	MetS (*n* = 49)	*p*
Length of gestation, weeks	39.4 ± 1.3	39.2 ± 1.3	0.30
Weight gain in pregnancy, kg	17.0 ± 6.8	16.5 ± 6.3	0.64
Male infant	522 (52.9)	24 (52.2)	0.93
Caesarean delivery	380 (38.6)	27 (61.4)	0.003
Birthweight, g	3286 ± 451	3383 ± 498	0.19
LGA	116 (11.8)	7 (15.2)	0.48
Outcomes associated with CV risk:			
SGA	69 (7.0)	2 (4.4)	0.76
Preterm delivery	42 (3.7)	3 (6.1)	0.43
Gestational diabetes	24 (2.1)	1 (2.0)	0.99
Preeclampsia	14 (1.2)	0 (0.0)	0.99

Categorical variables shown as *n* (%). Denominators vary for some variables due to missingness.

CV, cardiovascular; LGA, large-for-gestational age; SGA, small-for-gestational age.

## Results

A total of 1183 women who had a subsequent singleton pregnancy underwent systematic assessment of cardiovascular risk factors at median 24.7 weeks before pregnancy. The women were stratified into two groups based on their pregravid assessment: those who met criteria for MetS (*n* = 49) and those who did not (Table 1). There were no significant differences in age, years of education, smoking status, passive smoking exposure, preexisting hypertension, or preexisting diabetes between women who met criteria for MetS and their peers. As expected, each MetS component differed between the groups. Specifically, waist circumference, systolic blood pressure, diastolic blood pressure, triglycerides, and fasting glucose were significantly higher in the MetS group, while HDL cholesterol was significantly lower (all *p* < 0.0001). The mean BMI of women without MetS was 20.2 kg/m^2^, while that of women who met criteria for MetS was 23.1 kg/m^2^ (*p* < 0.0001). Women with pregravid MetS also had significantly higher LDL cholesterol levels than their peers (2.4 mmol/L vs. 2.0 mmol/L, *p* = 0.02). There was no difference between the groups in total cholesterol.

We next compared pregnancy outcomes between women with and without pregravid MetS (Table 2). There were no significant differences in length of gestation, weight gain in pregnancy, infant sex, birthweight, or prevalence of LGA infants between women who met criteria for MetS before pregnancy and their peers. Women with MetS were more likely to have a Caesarian section than their peers (*p* = 0.003). Of note, however, women with pregravid MetS were no more likely than women without to experience GDM, preeclampsia, preterm delivery, or delivery of an SGA infant.

We next considered whether the women who developed *any* of these four pregnancy outcomes associated with future CVD showed evidence of an adverse cardiovascular risk factor profile before pregnancy. These women (*n* = 141) did not have a higher prevalence of pregravid MetS than that of their peers (4.3% vs. 4.5%, *p* = 0.92). Similarly, they did not have a higher prevalence before pregnancy of any of the MetS component disorders (Fig. 1). Indeed, their pregravid cardiovascular risk factor profile was unremarkable when compared to that of their peers (Fig. 2). Specifically, there were no significant differences in pregravid waist circumference, BMI, blood pressure, HDL cholesterol, triglycerides, LDL cholesterol, or fasting glucose between women who subsequently developed any of the future CVD-associated pregnancy outcomes and those who did not.

## Discussion

In this study, we have evaluated the relationship between the maternal pregravid cardiometabolic risk factor profile and four pregnancy outcomes that identify an increased risk of CVD later in life: GDM, preeclampsia, preterm delivery, and SGA infant in a low-risk population of young, nulliparous women. We show that the women with pregravid MetS were more likely to have a Caesarean delivery, but otherwise did not differ from their peers in pregnancy outcomes. In particular, women with MetS before pregnancy were no more likely than their peers to experience GDM, preeclampsia, preterm delivery, or delivery of an SGA infant. Similarly, women who had at least one of these four adverse pregnancy outcomes did not have a higher prevalence of MetS or any of its component disorders before pregnancy, compared to women who experienced no adverse pregnancy event. It thus emerges that the adverse cardiovascular risk factor profile that is seen in women with a history of GDM, preeclampsia, preterm delivery, or SGA infant does not necessarily manifest before their pregnancy.

It is well-established that certain adverse pregnancy events are predictive of future CVD and CVD mortality, although the precise mechanism underlying this association remains unclear. One prevailing theory proposes that the physiological stress of pregnancy unmasks a woman's preexisting cardiometabolic dysfunction, thus resulting in pregnancy complications and the accrual of cardiovascular risk factors over time that ultimately lead to CVD.^26^ This model is supported by the finding that many women with a history of adverse pregnancy outcomes exhibit an adverse cardiovascular risk factor profile within the first few months to years after delivery—potentially reflecting too short of an interval for cardiometabolic dysfunction to be solely the result of the pregnancy itself. Indeed, by as early as 3 months postpartum, women with a history of GDM have a higher prevalence of MetS, hypertension, dyslipidemia, elevated C-reactive protein, and hypoadiponectinemia.^27–29^ Similarly, at 1 year postpartum, the prevalence of MetS is higher in women with a history of preeclampsia, compared to their peers.^30^ By 2.5 years postpartum, women who experienced a hypertensive disorder of pregnancy have, on average, higher BMI, waist circumference, total cholesterol, and triglycerides, as well as a higher prevalence of hypertension and MetS.^21^ Thus, the presence of cardiovascular risk factors in the early postpartum period raises the question of whether an adverse cardiometabolic risk factor profile may be present before pregnancy in women who subsequently experience these adverse pregnancy outcomes.

Few previous studies have explored the relationship between prepregnancy cardiometabolic characteristics and adverse pregnancy outcomes. In the Coronary Artery Risk Development in Young Adults (CARDIA) study, Catov et al. linked self-reported pregnancy data to maternal lipid levels that were measured an average of 6 years before pregnancy.^31^ They noted a U-shaped relationship between prepregnancy total cholesterol and risk of preterm delivery.^31^ In another analysis from the CARDIA study, prepregnancy impaired fasting glucose, elevated fasting insulin, and low HDL cholesterol measured at median 33.6 months before pregnancy predicted 154 cases of self-reported GDM in 141 women.^32^ Magnussen et al. linked national birth registry data to a Norwegian population-based cohort, in which maternal lipid levels were assessed an average of 3 to 4 years before delivery.^33^ Positive independent associations were reported between subsequent preeclampsia and certain pregravid maternal characteristics, including levels of triglycerides, total cholesterol, LDL cholesterol, and blood pressure.^33^ Thus, taken together, previous studies that evaluated the relationship between pregravid maternal health and subsequent pregnancy outcomes have been collectively limited by risk factor profiling performed at ∼3 to 6 years before the pregnancy and self-reported outcomes.

The current prospective preconception cohort aimed to address this question in a low-risk population of young, nulliparous women. By recruiting newly married women who were planning to conceive in the coming months, we were able to follow such a population of women prospectively from prepregnancy to delivery and limit the interval between pregravid cardiometabolic characterization and the index pregnancy to a median of 24.7 weeks. Other strengths of this design are the prospective ascertainment of the pregnancy outcomes of interest and the comprehensive pregravid cardiovascular risk factor profiling, in which all components of MetS, as well as BMI and LDL cholesterol, were evaluated.

A limitation of this study is that the reasons for Caesarean section and preterm delivery were not available. Second, because the study population was young, lean, and healthy, only 4.1% of the participants met criteria for pregravid MetS. Furthermore, the cohort consisted of Chinese women from one region in the province of Hunan. Therefore, the generalizability of these results to older, less healthy women, as well as to women of other ethnicities, remains uncertain. That said, the design feature of recruiting women from the premarriage health clinics at the Liuyang Maternal and Infant Hospital was crucial for the cost-efficient development of a prospective preconception cohort. Finally, it is not known if the association between adverse pregnancy outcomes and future CVD is attenuated when the pregnancy outcome occurs in young adulthood, rather than later.

Our results did not show differences in pregravid cardiometabolic profiles between the women who subsequently experienced adverse pregnancy outcomes and those who did not, although further work is required to better understand this relationship. It is quite possible that the statistical power in the study was not sufficient to demonstrate a relationship between pregravid health and pregnancy outcomes in this low-risk population. In a less healthy population, a significant association between pregravid MetS and adverse pregnancy outcomes may be evident. Furthermore, it is possible that some women who subsequently experience adverse pregnancy outcomes have pregravid cardiometabolic dysfunction that is too subtle to detect using the measurements used in this study. With time, this cardiovascular and metabolic dysfunction may become more severe and eventually detectable. Indeed, it has recently been demonstrated that the adverse cardiovascular risk factor profile of women with a history of GDM begins to evolve over time in the years before the index pregnancy.^34^

Current guidelines encourage clinicians to monitor and address cardiovascular risk factors in the early postpartum period in women who have a history of adverse pregnancy outcomes.^2^ In this context, our study of a young, low-risk population is reassuring in showing that an adverse cardiovascular risk factor profile does not necessarily precede pregnancy in women who have these antepartum outcomes, thereby suggesting that medical and lifestyle interventions to mitigate future CVD risk can be potentially implemented after the pregnancy. As the pathophysiology underlying this relationship becomes better understood, it is possible that interventions may be appropriately timed to address the risk of cardiometabolic dysfunction in a timely manner.

## Conclusions

In this low-risk population of young women, those who had pregravid MetS were more likely to have a Caesarean delivery, but otherwise did not differ from their peers with respect to the likelihood of having GDM, preeclampsia, preterm delivery, or delivery of an SGA infant. Similarly, women who had at least one of these four adverse pregnancy outcomes did not have a higher prevalence of MetS or any of its component disorders before pregnancy. It thus emerges that the adverse cardiovascular risk factor profile that is seen in women with a history of GDM, preeclampsia, preterm delivery, or SGA infant does not necessarily manifest before pregnancy, suggesting that a window of opportunity for risk modification may be present in the years after delivery.

## Abbreviations Used

BMI: body mass index
CARDIA: Coronary Artery Risk Development in Young Adults
CV: cardiovascular
CVD: cardiovascular disease
GDM: gestational diabetes mellitus
HDL: high-density lipoprotein
LDL: low-density lipoprotein
LGA: large-for-gestational age
MetS: metabolic syndrome
SGA: small-for-gestational age
